# Metronidazole-Induced Drug Reaction with Eosinophilia and Systemic Symptoms (DRESS) Syndrome With Parvovirus B19 Reactivation: A Pediatric Case

**DOI:** 10.7759/cureus.62125

**Published:** 2024-06-11

**Authors:** Bahaeddine Dridi, Myriam Agrebi, Dhouha Sahnoun, Chaker Ben Salem

**Affiliations:** 1 Pharmacology, Pharmacovigilance Center of Sousse, Faculty of Medicine of Sousse, University of Sousse, Sousse, TUN; 2 Pharmacology, Faculty of Pharmacy of Monastir, University of Monastir, Monastir, TUN

**Keywords:** dress syndrome, drug reaction with eosinophilia and systemic symptoms, pharmacology, pharmaco-vigilance, pediatrics, parvovirus b-19, dress and viruses, metronidazole side effects

## Abstract

Drug reaction with eosinophilia and systemic symptoms (DRESS) is a severe and rare syndrome that causes life-threatening organ dysfunctions. Here, we present the case of a 10-year-old child who developed a pruritic erythematous eruption, fever, facial edema, and lymphadenopathy seven days after receiving intravenous metronidazole (20 mg/kg/day), vancomycin (50 mg/kg/day), and cefotaxime (200 mg/kg/day). Laboratory tests showed eosinophilia and liver damage as well as positive parvovirus B19 IgM and IgG indicating viral reactivation. Vancomycin was initially discontinued and later reintroduced with no ill effects. The patient was managed with topical corticosteroid emollients and cetirizine and improved within seven days of metronidazole withdrawal. Treatment with cefotaxime was continued and showed no adverse effects.

## Introduction

Drug rash with eosinophilia and systemic symptoms (DRESS) is a severe drug hypersensitivity reaction that causes life-threatening organ dysfunctions [1]. Its diagnosis is established using diagnostic criteria proposed by the International Registry of Severe Cutaneous Adverse Reactions (RegiSCAR), which employs a scoring system based on both clinical and laboratory findings [2].

DRESS syndrome in pediatric patients is uncommon, as evidenced by the limited case reports in the literature. Children with DRESS have significantly lower mortality of approximately 1% compared to 10% in adults [3]. Antibiotics, mainly beta-lactams (50%), are the most common cause of DRESS in children [4]. The standard treatment involves prompt discontinuation of the causative medicine along with corticosteroids, antihistamines, and close monitoring for potential complications.

Here, we present an extremely rare case of metronidazole-induced DRESS associated with parvovirus B19 reactivation. Based on our literature search, this appears to be the first reported case on these parameters.

## Case presentation

**Figure 1:**
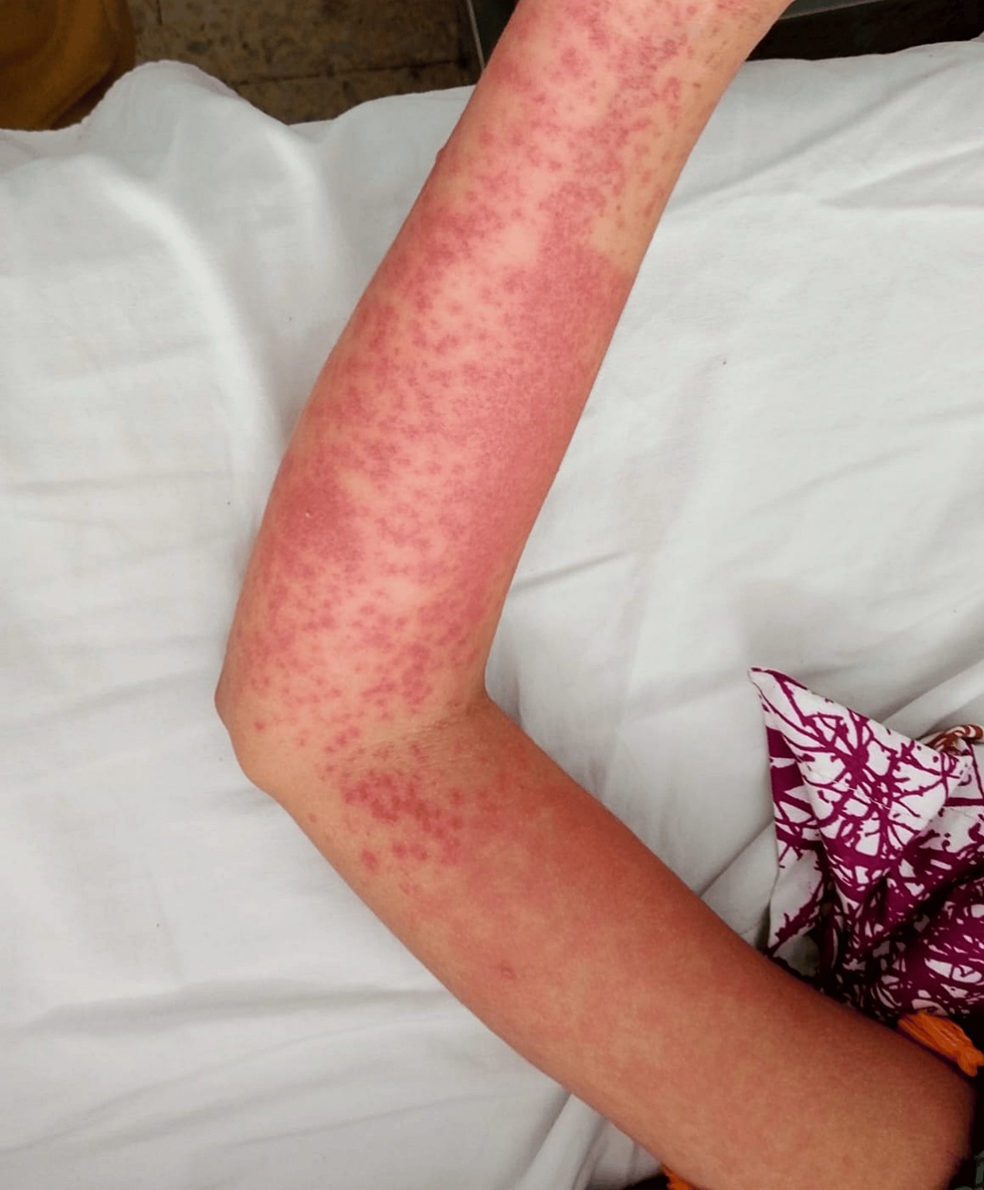
Diffuse morbilliform rash over the arms

A 10-year-old girl was referred to the Department of Pediatrics due to a para-pharyngeal abscess. On interviewing, we found that she had a tonsillectomy five years ago, had no prior history of drug hypersensitivity, and did not take any medications prior to hospitalization. On admission, treatment was initiated with cefotaxime (200 mg/kg/day), vancomycin (50 mg/kg/day), and intravenous metronidazole (20 mg/kg/day). Seven days later, she developed a diffuse pruritic and erythematous eruption. Physical examination revealed a fever (39°C), facial edema, lymphadenopathy, and a diffuse maculopapular rash covering 80% of the body surface located on the thorax, upper and lower limbs, trunk, and back (Figure 1).

Laboratory tests conducted on the same day showed eosinophilia (eosinophils count 800/mm³), mild cytolysis, (aspartate aminotransferase: 60 U/L, alanine aminotransferase: 55 U/L), elevated gamma-glutamyl transferase at 90 U/L and a mild increase of C-reactive protein of 11 mg/L. Serology for chlamydia trachomatis and mycoplasma were negative. Similarly, viral serologies for hepatitis, cytomegalovirus (CMV), Epstein-Barr virus (EBV), and human herpes virus 6 (HHV-6) were negative. However, parvovirus IgM and IgG were positive, which was consistent with the reactivation of parvovirus B19. Skin biopsy showed a spongiosis in the epidermis with mild lymphocytic infiltration in the upper dermis which are often observed in the histopathology of DRESS syndrome but are not specific to it.

Based on the clinical and laboratory features, DRESS was suspected. Application of the Registry of Severe Cutaneous Adverse Reaction (RegiSCAR) DRESS criteria yielded a score of 5 which means this is a “probable case” (Table 1) [2]. The Naranjo Adverse Drug Reaction score for metronidazole was 3, indicating a “possible case” [5].

**Table 1:** Results of the RegiSCAR scoring system * Final score < 2: no case, final score 2-3: possible case, final score 4-5: probable case, and final score > 5: definite case. ANA, anti-nuclear antibody; BSA, body surface area; HAV, hepatitis A virus; HBV, hepatitis B virus; HCV, hepatitis C virus; DRESS, drug reaction with eosinophilia and systemic symptoms; RegiSCAR, Registry of Severe Cutaneous Adverse Reactions

Items	Scoring for DRESS			Patient’s results	Patient’s score
	Yes	No	Unknown		
Fever ≥ 38.5°C	0	-1	-1	Yes	0
Enlarged lymph nodes	1	0	0	Yes	1
Eosinophilia ≥ 0.7 x 10^9^/L	1	0	0	Yes	1
Eosinophilia ≥ 1.5 x 10^9^/L or ≥ 20%	2			No	
Atypical lymphocytes	1	0	0	No	0
Skin rash > 50% BSA	1	0	0	Yes	1
Rash suggesting DRESS	1	-1	0	Yes	1
Skin biopsy suggesting DRESS	0	-1	0	Yes	0
Organ involvement (score 1 for each organ, maximal score:2)	1	0	0	Yes	1
	2			No	
Rash resolution ≥ 15 days	0	-1	-1	No	-1
Excluded other causes (≥ 3 tests of the following tests were negative: HAV, HBV, HCV, Mycoplasma, Chlamydia, ANA, Blood culture)	1	0	0	Yes	1
Final Score*					5

Vancomycin was the first to be discontinued following our thorough literature review, which highlighted its frequent association with DRESS syndrome compared to cefotaxime or metronidazole. However, we noted a worsening of pruritus and rash. Three days later, on hospital day 10, we decided to withdraw metronidazole while cefotaxime was continued and vancomycin was reintroduced at 50 mg/kg/day. After three days, on hospital day 13, the patient gradually started to improve with a resolution of the swelling of the face and lymph nodes and the skin rash.

Along with medicine withdrawal, the patient was managed with topical betamethasone 0.05% and cetirizine 10 mg/day for 14 days. Systemic corticosteroids were deemed unnecessary as the patient displayed no signs of severity. Complete recovery of the fever, skin rash, and laboratory abnormalities was observed on hospital day 17 meaning one week after metronidazole withdrawal and the patient was discharged on amoxicillin/clavulanic acid (1 g/day), which was prescribed for five days and showed no adverse events. One month later, on follow-up, the patient was completely asymptomatic and had no relapse of the rash. Five months after the complete resolution of symptoms, a skin patch test for metronidazole was performed with negative results.

## Discussion

Metronidazole was identified as the causative drug due to the clear temporal relationship between its administration and the onset of symptoms, along with the marked improvement observed three days after its discontinuation. The negative patch test was not in favor of the DRESS diagnosis but it does not exclude it. Indeed, patch test sensitivity in DRESS patients ranged from 32% to 64% [6]. There is no definite explanation for false negative results, but theoretically, there may be several possible causes: (a) the final responsible agent is a drug metabolite that is not formed in the skin during patch testing, (b) there is no immune mechanism involved, (c) concomitant factors that are responsible in inducing transient drug intolerance, such as viral infection, are not present at the time of testing, and (d) wrong choice of vehicle (limited skin penetration), drug concentration, or exposure time [7]. It is difficult to explain all the clinical features such as facial edema, biological abnormalities such as eosinophilia, and this RegiSCAR score by an isolated parvovirus B19 reactivation in the absence of a drug hypersensitivity syndrome.

Metronidazole is considered a well-tolerated drug which explains its widespread usage. The most common side effects are gastrointestinal disturbances and reversible hematological abnormalities [8]. Cutaneous adverse reactions attributed to metronidazole are rare. However, a variety of different reaction types have been reported including Stevens-Johnson/toxic epidermal necrolysis and acute generalized exanthematous pustulosis [9].

DRESS due to metronidazole has been reported in the literature in two possible cases [10,11]. DRESS generally has a long latency period, from two to six weeks after initiation of the offending drug [2]. However, in these cases and the present case, the onset of symptoms was rapid and occurred within one week. In 2012, Seto et al. described the first case of suspected metronidazole-induced DRESS occurring in a 67-year-old woman who experienced a fever and diffuse maculopapular rash, starting four hours after metronidazole intake following at least one previous sensitizing exposure [10]. Recently, in 2022, Soares et al. reported the second case of DRESS occurring in a 66-year-old male; seven days after receiving oral metronidazole, he recovered within eight days of the drug’s withdrawal [11].

The association between DRESS syndrome and viral infection is well documented and a specific contribution from the virus to DRESS symptoms and severity is possible but not yet established. It has been proposed that DRESS may essentially be a viral disease, triggered by the direct effect of drugs on viral reactivation [12]. DRESS syndrome can also occur when an immune response is formed against the drug, which results in a cytokine storm and a secondary viral reactivation could occur [4]. Patients with viral reactivation were found to have an increased number of relapses, a longer disease duration, and increased rates of hepatitis, renal failure, myocarditis, and death compared to DRESS patients with no evidence of viral reactivation [13]. Human herpes virus (HHV)-6, HHV-7, herpes simplex virus (HSV), CMV, and EBV reactivation have been associated with DRESS syndrome [12,14]. However, the most convincing data about the association between viral infection and DRESS syndrome concerns HHV-6. Indeed, HHV-6 reactivation is one of the diagnostic criteria for Drug-Induced Hypersensitivity Syndrome (the Japanese equivalent of DRESS) [15,16].

For the specific case of parvovirus B19, a nonenveloped single-stranded DNA virus of the *Parvoviridae* family, its presence in DRESS has been reported in a few cases. Coughlin et al. reported the cases of two pediatric patients (11- and 13-year-old boys) of DRESS that were associated with parvovirus B19 infection [17]. The first was admitted for chemotherapy treatment of acute myelogenous leukemia. He developed fever, eruption, facial edema, and lymphadenopathy 11 days after starting cefepime therapy. The second was admitted with fever, eruption, facial edema, and lymphadenopathy several months after initiation of lamotrigine. Recently, Cura and colleagues also reported five cases of DRESS associated with a positive polymerase chain reaction (PCR) study for parvovirus B19 DNA in the skin samples of four patients and in the blood sample of an adult female treated with clotiapine for depression [18].

It is important to mention that viral infections are a common cause of skin rashes in children and viral rashes may mimic DRESS syndrome. Parvovirus B19 infection mimicking DRESS has been reported in a single case in the literature where the patient presented with a scarlatiniform eruption associated with multiple node enlargement, elevated liver enzymes, and an abnormal white cell count with positive parvovirus B19 PCR and without any history of drug intake [19].

## Conclusions

This case highlights the possibility of metronidazole-induced DRESS syndrome. While not conclusively proven, cases like this prompt important discussions surrounding this syndrome, its diagnosis, and the identification of potential triggering drugs. Our case also shows that parvovirus B19 reactivation may be implicated in DRESS syndrome. More case studies are necessary to clarify the association between DRESS syndrome and this virus. Considering the wide use of metronidazole, physicians should be aware of the possibility of DRESS syndrome occurring during its use.
